# Mitochondrial protein 18 (MTP18) plays a pro-apoptotic role in chemotherapy-induced gastric cancer cell apoptosis

**DOI:** 10.18632/oncotarget.17508

**Published:** 2017-04-28

**Authors:** Lynn H.H. Aung, Ruibei Li, Bellur S. Prabhakar, Ajay V. Maker, Peifeng Li

**Affiliations:** ^1^ Department of Microbiology and Immunology, College of Medicine, University of Illinois at Chicago, Chicago, IL, USA; ^2^ Division of Surgical Oncology, Department of Surgery, University of Illinois at Chicago, Creticos Cancer Center, Advocate Illinois Masonic Medical Center, Chicago, IL, USA; ^3^ School of Professional Studies, Northwestern University, Chicago, IL, USA

**Keywords:** MTP18, mitochondrial fission, apoptosis, chemotherapy, gastric cancer

## Abstract

One of the severe limitations of chemotherapy is the development of drug resistance. However, the mechanisms underlying chemotherapy resistance remain to be elucidated. Mitochondrial mediated apoptosis is a form of cell death induced by chemotherapy. Several chemotherapeutic agents have been shown to induce mitochondrial fission, and finally activate the apoptosis cascade in various cancer cells. Here, we report that the mitochondrial membrane protein 18 (MTP18) induced mitochondrial fragmentation in gastric cancer cells under doxorubicin (DOX) exposure. Upon over-expression of MTP18, a sub-cytotoxic dose of DOX could sensitize a significant number of cells to undergo mitochondrial fission and subsequent apoptosis. These findings suggest that MTP18 can enhance the sensitivity of gastric cancer cells to DOX. Mechanistically, we found that MTP18 enriched dynamic-related protein 1 (DRP1) accumulation in mitochondria and it was responsible for mediating DOX-induced signaling required for mitochondrial fission. Intriguingly, MTP18 expression was downregulated during DOX treatment. Thus, down-regulation of MTP18 expression could be one of the resistance factors interfering with DOX-induced apoptosis in gastric cancer cells.

## INTRODUCTION

Gastric cancer is the fifth most common cancer and the third leading cause of cancer-related deaths worldwide [1]. Chemotherapy plays a crucial role in gastric cancer management especially for advanced stages [2–4]. Yet, one of its greatest obstacles in achieving successful outcome is the development of chemoresistance [5, 6]. Hence, a deeper understanding of the molecular basis of resistance to chemotherapy is critical to uncover novel approaches to enhance chemosensitivity in gastric cancer cells.

Doxorubicin (DOX), an anthracycline chemotherapeutic agent that functions by DNA intercalation, has been widely used in various cancer treatment regimens [7–9]. It has been successfully used as a single-agent in the treatment of gastric adenocarcinoma, and along with the other anthracycline chemotheraputics in multiple combination gastric cancer chemotherapy regimens, including FAMTX, M-FAMTX, EAP, ECF, and PELF [10–13]. Apoptosis is a form of cell death induced by DOX in cancer cells; DOX conveys its apoptotic signal through mitochondrial pathway [14, 15]. Additionally, our previous studies using in-vitro as well as in-vivo gastric cancer models showed that DOX-induced apoptosis is mediated through mitochondrial fission [16, 17]. However, the exact molecular mechanism by which DOX-induced mitochondrial fission mediated apoptosis is not fully understood.

In addition to the conventional role of major energy generator of the cell, mitochondria serve as the active regulator of apoptotic cascades [18–21]. Studies have reported that mitochondrial fission is involved in the initiation of apoptosis, while mitochondrial fusion is able to inhibit apoptosis [22, 23]. Fragmentation of mitochondria increases the mitochondrial membrane permeability, and initiates the release of mitochondrial pro-apoptotic factors such as cytochrome c (Cyt-c), which further activates the downstream apoptotic cascades [24, 25]. Mitochondrial outer membrane proteins including DRP1 and mitochondrial fission 1 protein (Fis1) are known to be the key mediators of mitochondrial fission [26, 27]. Nevertheless, very little is known about the potential involvement of mitochondrial inner membrane proteins in the regulation of mitochondrial fission machinery.

A novel Mitochondrial Protein 18 (MTP18), also known as Mitochondrial Fission Protein 1 (MTFP1), was first demonstrated to implicate in the regulation of mitochondrial fission in human prostate cancer (PC-3) cells and human keratinocytes (HeCaT) [28]. MTP18 is a mitochondrial inner membrane protein and has a molecular size of 18kDa [28]. It is encoded by a nuclear PI-3 kinase dependent target gene (GenBank accession number AAH46132). MTP18 serves as a down-stream effector of the PI-3 kinase signaling pathway. MTP18 is essential for mitochondrial viability by maintaining the integrity of the mitochondrial network [29]. However, it remains unknown as to whether MTP18 can affect the chemotherapy-induced mitochondrial fission and/or apoptosis, and thus can be related to chemoresistance. Therefore, this study aimed to understand the role of MTP18 in chemotherapy-induced mitochondrial fission and apoptosis and to elucidate by which mechanism MTP18 is involved in these processes.

We used doxorubicin (DOX) as an apoptotic inducer in this study due to the following reasons: 1) DOX is one of the most commonly used chemotherapeutic agents in treating gastric cancer, and 2) several lines of evidence showed that it can induce mitochondrial pathway of apoptosis [7–9]. Our results showed that DOX-induced gastric cancer cells apoptosis can be regulated by MTP18. MTP18 is able to enhance mitochondrial fission and apoptosis in gastric cancer cells by promoting DRP1 accumulation in mitochondria upon DOX treatment. Enforced expression of MTP18 significantly increases the sensitivity of gastric cancer cells to DOX. Our data suggests that MTP18 can increase the chemosensitivity in gastric cancer by targeting mitochondrial fission machinery.

## RESULTS

### Doxorubicin induces apoptosis and mitochondrial fission in gastric cancer cells

In order to explore the role of MTP18 in DOX-induced apoptosis, we determined the extent of doxorubicin induced apoptosis in gastric cancer AGS cells by detecting the levels of cleavage of procaspases including caspase-3 and PARP1. The cells were treated with different concentrations of DOX or a single concentration for different durations. We observed a time- and dose-dependent increase in cleavage of caspase-3 and PARP1 upon DOX exposure (Supplementary Figure 1A and 1B). Concomitantly, cell death ELISA results showed a time-dependent increase in apoptosis related DNA fragmentation upon DOX exposure (Supplementary Figure 1C).

**Figure 1:**
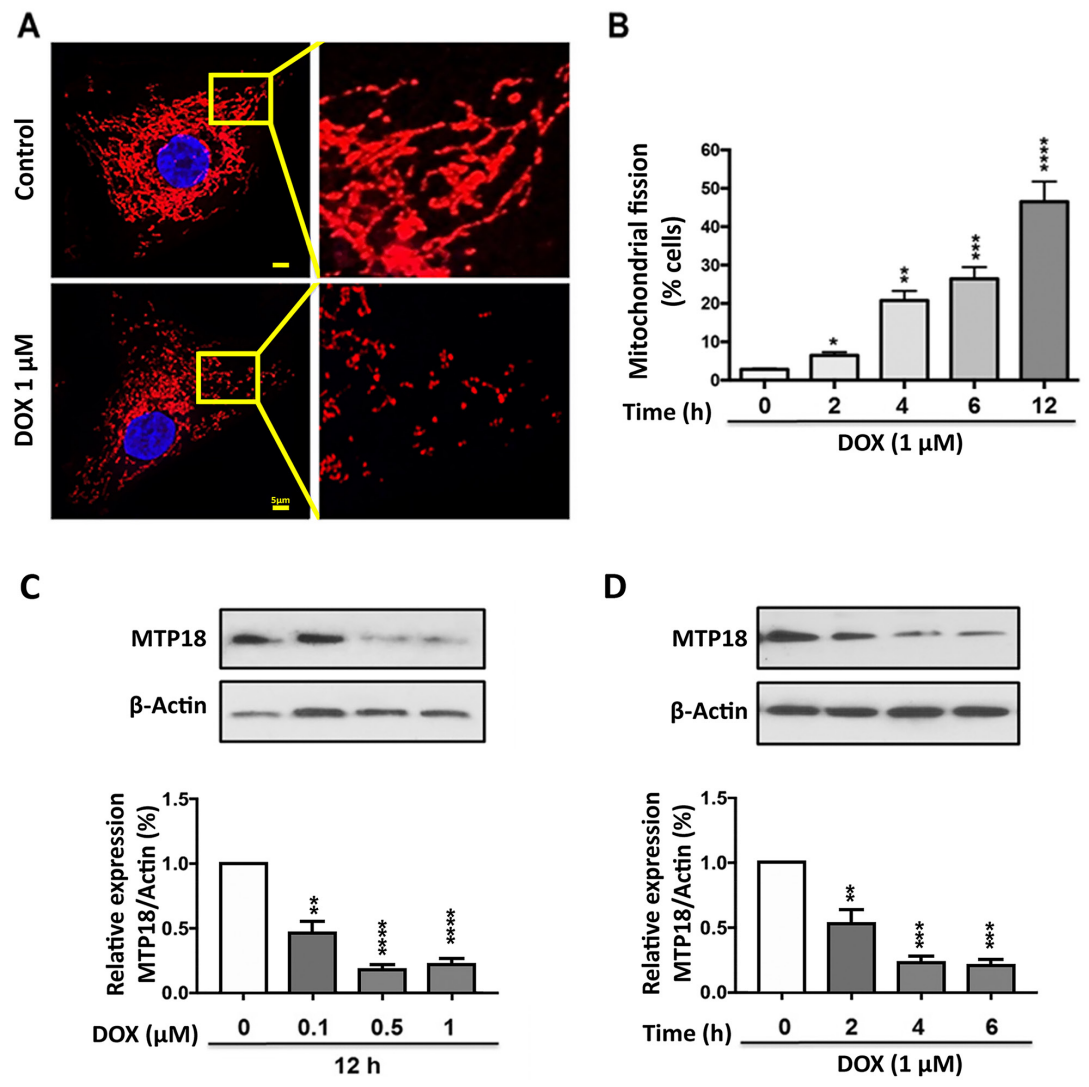
Doxorubicin exposure induces mitochondrial fission and down-regulates the MTP18 expression in a dose- and time- dependent manner **(A)** and **(B)** AGS cells were stimulated with 1μmol/L doxorubicin (DOX) at indicated time points and mitochondrial morphology was analyzed. **(A)** Shows mitochondrial morphology of control and after 6hr of DOX exposure. **(B)** Shows percentage of cells undergone mitochondrial fission upon DOX exposure. **(C)** and **(D)** Analysis of mitochondrial protein 18 (MTP18) expression. AGS cells were stimulated with the indicated doses of DOX and harvested at 12h **(C)**, and cells were stimulated with 1μmol/L DOX and then harvested at the indicated time **(D)** for immunoblotting. **(C)** and **(D)** *Upper panels* show mitochondrial protein 18 (MTP18) expression on DOX exposure. **(C)** and **(D)** *Lower panels* show densitometry analysis. β-actin served as a loading control. The densitometry data were expressed as the mean±SEM of three independent experiments. *P<0.05, **P<0.01, ***P<0.001, and ****P<0.0001 compared to non-treated control. Figures presented are the representative of at least three independent experiments.

Several studies reported that DOX could induce mitochondrial fission in various cancer cells [16, 17, 30]. To confirm that, we treated the AGS cells with DOX (1μmol/L) at different time points and counted the cells undergoing mitochondrial fission; we observed a time dependent increase in percentages of cell undergoing mitochondrial fission upon DOX exposure (Figure 1A and 1B).

### Mtp18 was downregulated upon DOX exposure in gastric cancer cells

Concurrently, we evaluated the expression levels of MTP18 in AGS cell lines upon DOX exposure with immunoblot. Our results showed DOX treatment caused a significant decrease in MTP18 expression in a time- and dose- dependent manner (Figure 1C and 1D), suggesting that MTP18 may play a role in the regulation of DOX-induced mitochondrial fission and apoptosis.

### Knockdown of MTP18 interferes with DOX-induced mitochondrial fission as well as apoptosis

**Figure 2:**
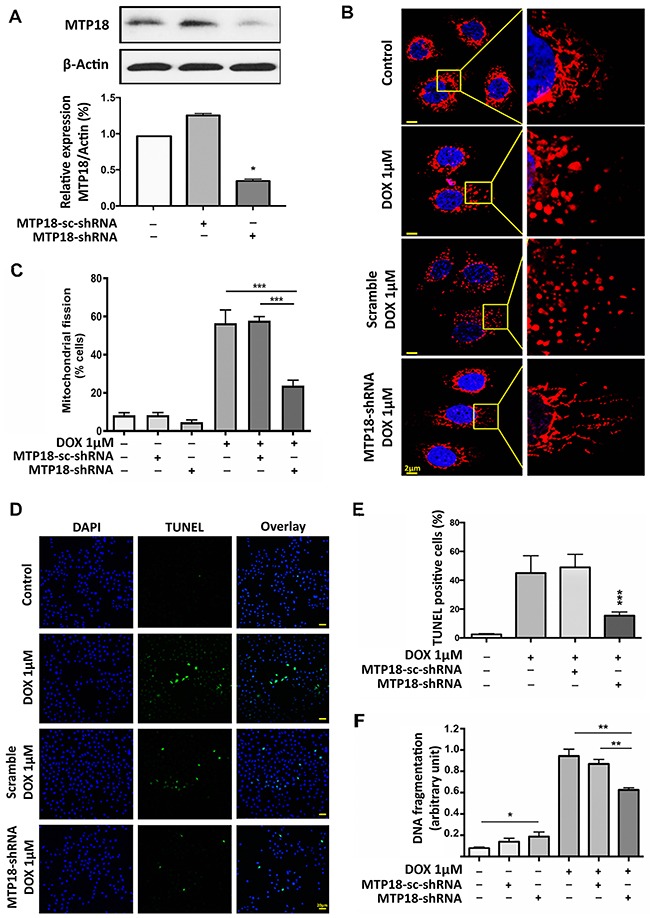
Knockdown of MTP18 interferes doxorubicin-induced mitochondrial fission and apoptosis **(A)** Analysis of mitochondrial protein 18 (MTP18) expression. Immunoblot shows MTP18 knockdown in AGS cells (*upper panel*). β-actin served as a loading control. The densitometry data were expressed as the mean±SEM of three independent experiments (*lower panel*). *P < 0.05 verses negative control or scramble (sc-shRNA). B and C. Knockdown of MTP18 interferes cells to undergo DOX-induced mitochondrial fission. AGS cells were exposed to high concentration (1μmol/L) doxorubicin (DOX). **(B)** Shows mitochondrial morphology. Bar=2μm. C shows percentage of cells with mitochondrial fission; ***P<0.001. **(D)** and **(E)** Knockdown of MTP18 interferes cells to undergo DOX-induced apoptosis. Apoptosis was analyzed by TUNEL assay. (D) Shows TUNEL positive cells. Bar=20μm. E shows percentage of TUNEL positive. **(F)** Knockdown of MTP18 interferes cells to undergo DOX-induced DNA fragmentation. DNA fragments were analyzed using the cell death detection ELISA. **(D-F)** AGS cells were exposed to high concentration (1μmol/L) DOX. *P<0.05, **P<0.01, and ***P<0.001 versus DOX alone or sc-shRNA treated with DOX. Data were expressed as the mean±SEM of three independent experiments. Figures presented are the representative of at least three independent experiments.

To understand whether MTP18 is important for DOX-induced apoptosis, we knocked down the endogenous expression of MTP18 using MTP18-shRNA. MTP18 expression levels were significantly reduced by its shRNA but not its scramble form (Figure 2A). Knockdown of MTP18 significantly reduced percentage of cells undergoing mitochondrial fission compared to its negative or scramble controls even with high concentration (1μmol/L) of DOX exposure (Figure 2B and 2C). At the same time, we observed a significant reduction in DOX-induced apoptosis in MTP18 knockdown group, as observed by TUNEL assay (Figure 2D and 2E). To further confirm the effect of MTP18 knockdown on apoptosis, we employed cell death ELISA assay and found that there was a consistent reduction in the DOX-induced DNA fragmentation in MTP18 knockdown group compared to its controls (Figure 2F). These data suggest that MTP18 may play a pro-mitochondrial fission and pro-apoptotic role in DOX-induced apoptosis.

### Overexpression of MTP18 sensitizes the gastric cancer cells to doxorubicin-induced mitochondrial fission and apoptosis

**Figure 3:**
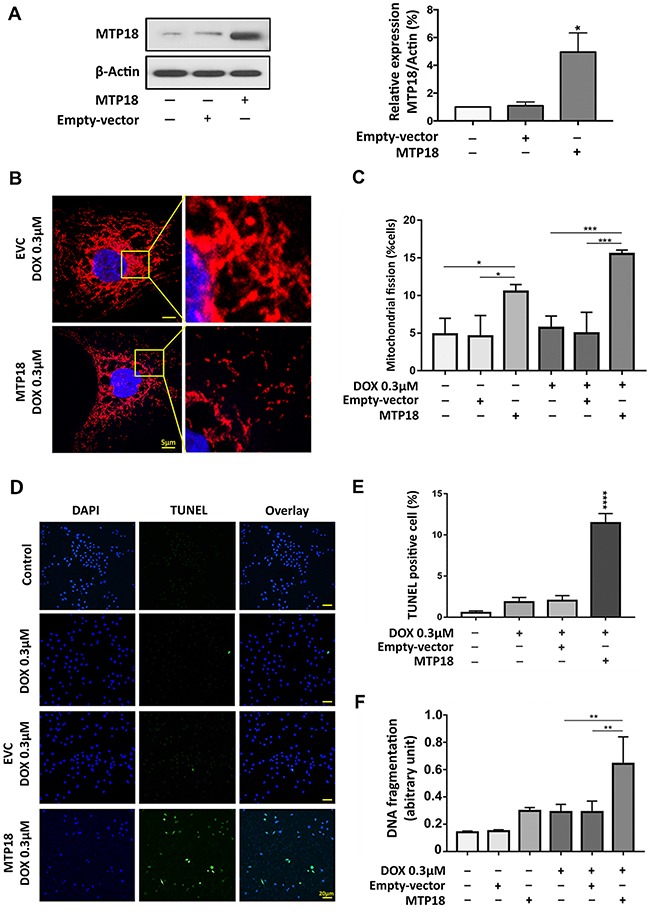
Enforced expression of MTP18 sensitizes doxorubicin-induced mitochondrial fission and apoptosis in AGS cells **(A)** Analysis of MTP18 expression. Immunoblot shows mitochondrial protein 18 (MTP18) overexpression in AGS cells (*left panel*). β-actin served as a loading control. The densitometry data were expressed as the mean ± SEM of three independent experiments (*right panel*). *P<0.05 verses negative control or empty vector control (EVC). **(B)** and **(C)** Enforced expression of MTP18 sensitizes cells to undergo DOX-induced mitochondrial fission. AGS cells were exposed to low concentration DOX. B shows mitochondrial morphology. Bar=5μm. (C) Shows percentage of cells with mitochondrial fission; *P<0.05, and ***P<0.001. **(D)** and **(E)** Enforced expression of MTP18 sensitizes cells to undergo DOX-induced apoptosis. Apoptosis was analyzed by TUNEL assay. **(D)** Shows TUNEL positive cells. Bar=20μm. **(E)** Shows percentage of TUNEL positive cells. **(F)** Enforced expression of MTP18 sensitizes cells to undergo DOX-induced DNA fragmentation. DNA fragments were analyzed using the cell death detection ELISA. **(D-F)** AGS cells were exposed to low concentration (0.3μmol/L) DOX. **P<0.01, and ****P<0.0001 versus DOX alone or empty vector control treated with DOX. Data were expressed as the mean ± SEM of three independent experiments. Figures presented are the representative of at least three independent experiments.

To augment the pro-mitochondrial function of MTP18, over expression of MTP18 in AGS cells was induced using lentiviral based MTP18-cDNA. The MTP18-cDNA significantly increased MTP18 expression in AGS cells as observed by immunoblot (Figure 3A). We found that a low concentration DOX (0.3 μmol/L) can induce a significantly higher number of cells to undergo mitochondrial fission in overexpressed group compared to its control groups, which show no significant change in percentage of mitochondrial fission before and after exposure (Figure 3B and 3C). Next, to explore the effect of MTP18 on apoptosis, we treated the cells with low concentration of DOX and quantified the percentages of apoptotic cell with ELISA and TUNEL assay. We observed a suboptimal dose of DOX, which did not trigger apoptosis in negative and empty vector control, could sensitize a significantly higher number of gastric cancer cells to undergo apoptosis in overexpressed group (Figure 3D, 3E and 3F). These findings suggest that MTP18 is able to enhance DOX-induced apoptosis in gastric cancer cells. The reduction of MTP18 expression in gastric cancer cells during DOX exposure could be an unfavorable response.

### Prediction of a MTP18′s target protein

To explore the mechanism, we performed protein interaction analysis to predict the target protein exhibiting the highest possible functional correlation with MTP18 [31]. The string search output revealed that 10 proteins obtained a medium or high confidence score of interaction (≥0.400). Of which, the DRP1 (also known as DNM1L) acquired the highest confidence score (0.742) to interact with MTP18 (MTFP1; Supplementary Figure 2A and 2B). In addition to having highest interaction score, DRP1 is one of the critical components of mitochondrial fission machinery in various types of mammalian cells [16, 32, 33]. Therefore, we selected DRP1 for further study.

### DRP1 translocates to the mitochondria upon DOX exposure

**Figure 4:**
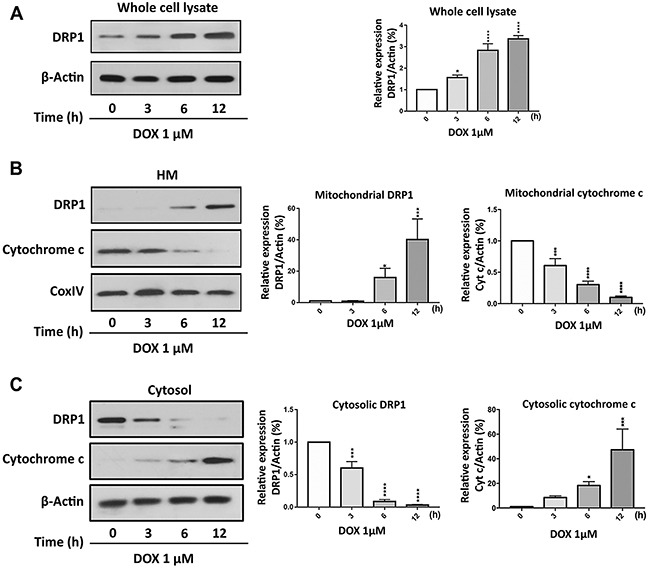
Doxorubicin exposure induces DRP1 accumulation in mitochondria **(A)** Doxorubicin (DOX) upregulated dynamic-related protein 1 (DRP1) expression in whole cell lysate. AGS cells were treated with 1μmol/L DOX and then harvested at the indicated time for immunoblot analysis of DRP1. **(B)** and **(C)** DRP1 translocates from cytosol to mitochondria, whereas cytochrome-c (Cyt-c) releases from mitochondria to cytosol upon DOX exposure. AGS cells were treated with 1μmol/L DOX and then harvested at the indicated time for immunoblot analysis of DRP1 and Cyt-c expression in the mitochondria and cytosol. **(A-C)** *Left panels* show immunoblot. **(A-C)** *Right panels* show densitometry analysis. HM= mitochondria-enriched heavy membranes. Cytochrome c oxidase (COXIV) served as a loading control for HM and β-actin served as a loading control for whole cell lysate and cytosolic fraction. Figures presented are the representative of at least three independent experiments. The densitometry data were expressed as the mean±SEM of three independent experiments. *P<0.05, ***P<0.001, and ****P<0.0001 versus non-treated control.

To understand the role of DRP1 in the regulation of DOX-induced apoptosis in gastric cancer cells, we initially tested the kinetic of DRP1 expression at different time points of DOX exposure. In Figure 4A, whole cell lysate analysis showed that DOX up-regulated the DRP1 expression. Others’ and our previous studies have shown that DRP1 translocates from the cytosol to mitochondria to initiate the mitochondrial membrane fragmentation upon exposure to cell stressors [16, 27, 33, 34]. To confirm that phenomenon in gastric cancer cells, we performed subcellular fractions and found that DRP1 was translocated to the mitochondria upon DOX exposure (Figure 4B and 4C) as observed by immunoblot. The accumulation of DRP1 in mitochondria further promoted the release of cytochrome c to cytosol (Figure 4B and 4C) and ultimately led to apoptosis [24].

### DRP1 is required for doxorubicin to induce mitochondrial fission and apoptosis

**Figure 5:**
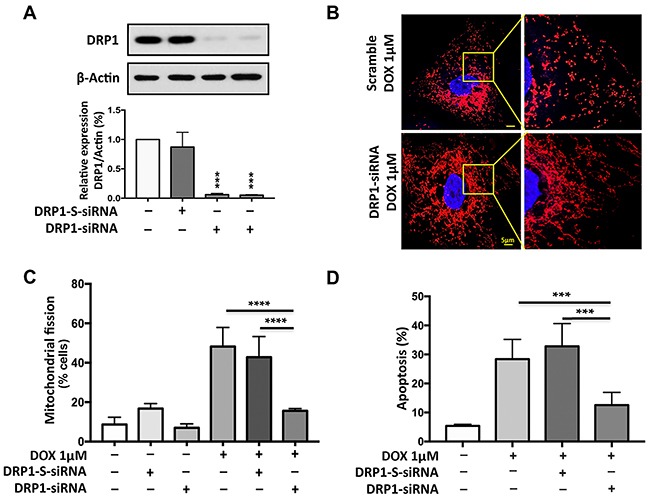
DRP1 is required for doxorubicin to induce mitochondrial fission and apoptosis **(A)** Analysis of DRP1 expression. Immunoblot shows dynamic-related protein 1 (DRP1) knockdown in AGS cells. Cells were transfected with 30 nM scramble siRNA, 15 nM (*lane 3*) or 30nM (*lane 4*) DRP1 siRNA (h) respectively and after 48hr, were harvested for immunoblot (*upper panel*). β-actin served as a loading control. The densitometry data were expressed as the mean±SEM of three independent experiments (*lower panel*). ***P<0.001 versus negative control or scramble (DRP1-S-siRNA). **(B)** and **(C)** Knockdown of DRP1 inhibits doxorubicin (DOX)-induced mitochondrial fission. B shows mitochondrial morphology. Bar=5μm. **(C)** Shows percentage of cells with mitochondrial fission; ****P<0.0001. **(D)** Knockdown of DRP1 inhibit DOX-induced apoptosis. Percentage of Apoptosis related DNA fragments was analyzed using the cell death detection ELISA. **(D)** Shows the percentage of apoptosis related DNA fragmentation; ***P<0.001. Figures presented are the representative of at least three independent experiments.

To test whether DRP1 is required for gastric cancer cell apoptosis, we knocked down the endogenous DRP1 expression by using DRP1-siRNA. DRP1-siRNA can significantly knockdown the expression level of DRP1 as analyzed by immunoblot (Figure 5A). Expectedly, upon treatment with high concentration (1μmol/L) of DOX, the DRP1 knockdown group showed a significantly lesser percentage of cells undergoing mitochondrial fission (Figure 5B and 5C) and apoptosis (Figure 5D) relative to that of scramble control as observed by mitochondrial staining and cell death ELISA assay.

### MTP18 promotes doxorubicin-induced DRP1 accumulation in the mitochondria

**Figure 6:**
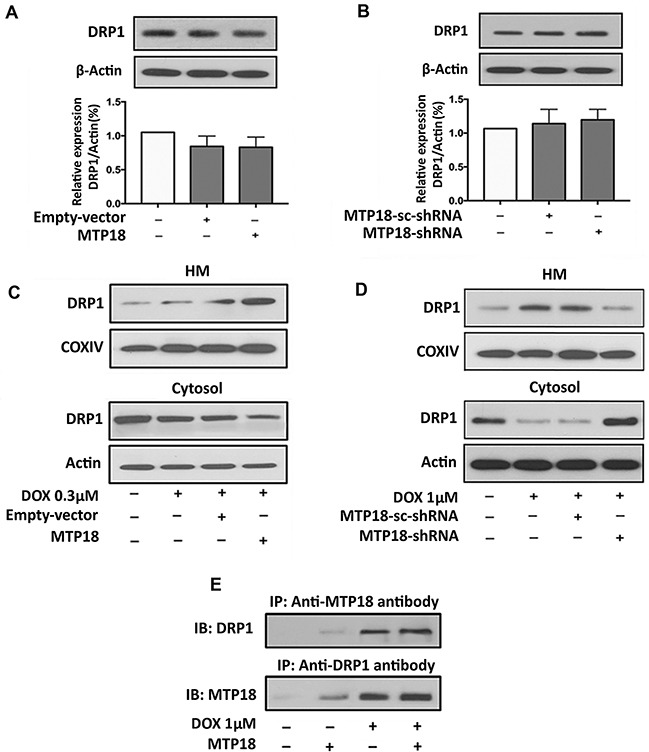
MTP18 promotes doxorubicin-induced DRP1 accumulation in mitochondria **(A)** and **(B)** Analysis of dynamic-related protein 1 (DRP1) expression. Immunoblot shows DRP1 expression in whole cell lysate upon modulation of mitochondrial protein 18 (MTP18) expression. Modulation of MTP18 does not significantly affect the expression level of DRP1 (*upper panel*). β-actin served as a loading control. The densitometry data were expressed as the mean±SEM of three independent experiments (*lower panel*). **(C)** and **(D)** Analysis of DRP1 expression in mitochondria and cytosolic fraction. Immunoblot shows DRP1 expression in AGS cells. C shows an increase in DRP1 accumulation in mitochondria upon enforced expression of MTP18. D shows a reduction in DRP1 accumulation when MTP18 was knocked down. HM= mitochondria-enriched heavy membranes. Cytochrome c oxidase (COXIV) served as a loading control for HM and β-actin served as a loading control for whole cell lysate and cytosolic fraction. **(E)** MTP18 binds to endogenous DRP1. Stable cell line of AGS infected with MTP18 were treated with 1μmol/L DOX. Since doxorubicin (DOX) exposure could induce a time-dependent downregulation of MTP18, the cells were harvested within 1h of DOX treatment to capture the association of MTP18 and DRP1 timely. The association between MTP18 and DRP1 was analyzed by immunoprecipitation (IP) followed by immunoblot (IB). Figures presented are the representative of at least three independent experiments.

To understand the functional correlation between MTP18 and DRP1, we first verified the level of DRP1 expression upon modulation of MTP18 expression. However, there is no significant change in the DRP1 expression levels regardless of enforced expression or knocking down of MTP18 (Figure 6A and 6B). It is of note that translocation of proteins during apoptosis allows the interaction of proteins in different cellular compartment [35, 36]. Therefore, we wanted to understand whether DRP1, translocates to mitochondrial outer membrane from cytosol upon cell stress can interact with MTP18, which is localized in mitochondrial inner membrane in chemotherapy-induced apoptosis. Interestingly, on subcellular fraction analysis, we found that overexpression of MTP18 promoted DRP1 accumulation in the mitochondria upon exposure to low dose DOX (Figure 6C, and Supplementary Figure 3A), which does not cause a significant change in mitochondrial DRP1 accumulation in negative control and empty vector groups. In contrast, when MTP18 was knocked down, even high concentration of DOX exposure could not induce a significant DRP1 translocation to mitochondria as compared to its negative control and scramble group (Figure 6D, and Supplementary Figure 3B). These data indicate that MTP18 might associate with DRP1 upon DOX exposure. To confirm their association, we performed the Immunoprecipitation (IP) using both MTP18 and DRP1 antibodies (Figure 6E). Within 1hr of DOX exposure, both MTP18 and DRP1 antibodies can pulldown MTP18 and DRP1 complex upon DOX exposure but not in the non-treated control. An increase in formation of DOX-induced MTP18 and DRP1 protein complex was observed, when MTP18 is overexpressed. These data indicated that MTP18 is required for mitochondrial DRP1 accumulation in the early phase of DOX exposure.

### MTP18 requires DRP1 to induce mitochondrial fission and apoptosis during doxorubicin exposure

**Figure 7:**
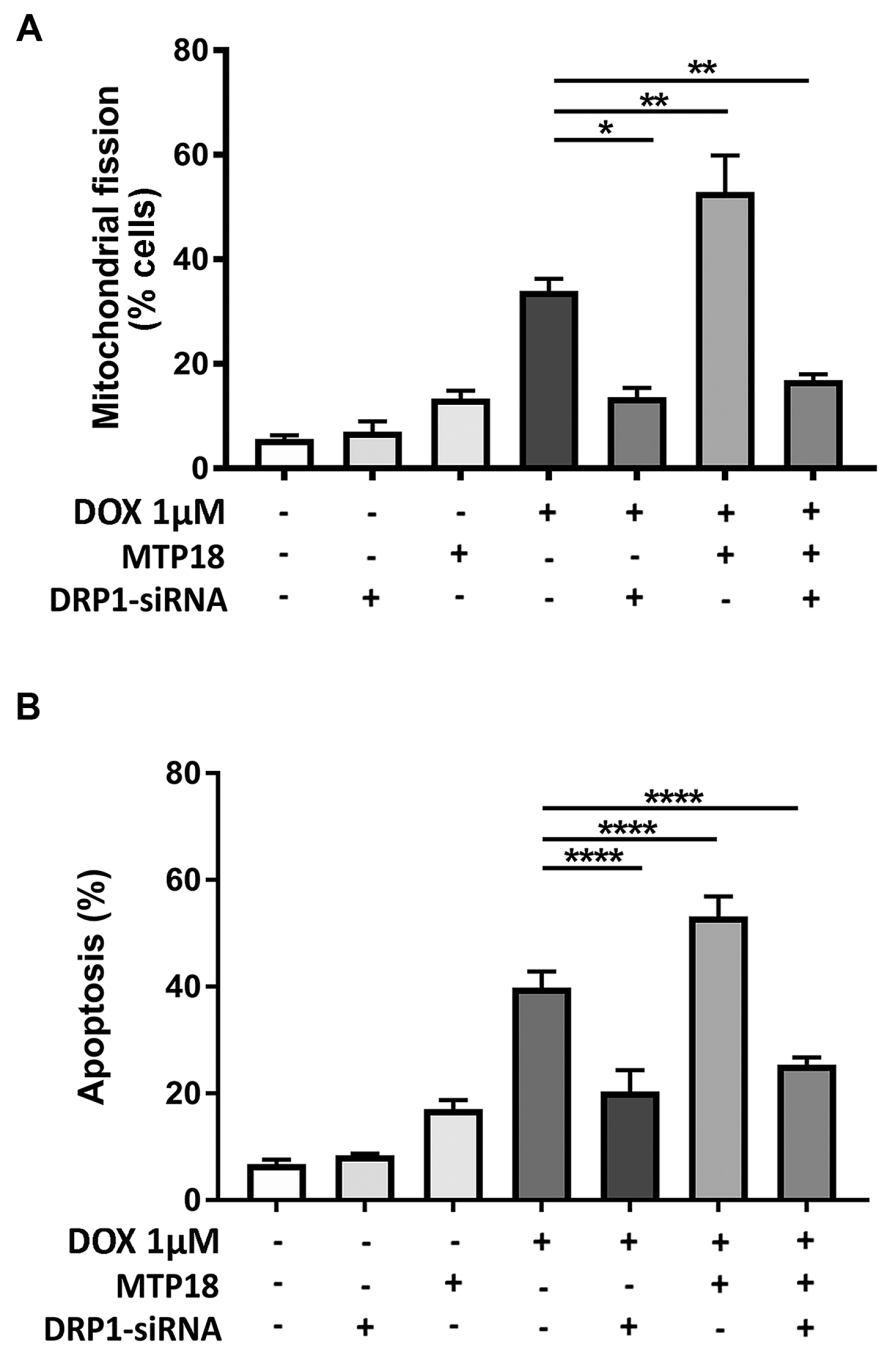
MTP18 requires DRP1 to induce mitochondrial fission and apoptosis during doxorubicin exposure **(A)** Mitochondrial protein 18 (MTP18)-induced mitochondrial fission upon doxorubicin (DOX) exposure is abolished when dynamic-related protein 1 (DRP1) is knocked down. Stable AGS cell line of overexpressed MTP18 was co-transfected with DRP1-siRNA and then treated with 1μmol/L DOX and percentages of cell undergoing mitochondrial fission was analyzed at 6hr. (A) Shows percentage of cells with mitochondrial fission. **(B)** MTP18-mediated apoptosis upon DOX exposure is abolished when DRP1 is knocked down. Stable AGS cell line of overexpressed MTP18 was co-transfected with DRP1-siRNA and treated with 1μmol/L DOX for apoptosis analysis at 24hr. The percentages of apoptosis were analyzed by cell death detection ELISA. (B) Shows percentages of apoptosis. Data were expressed as the mean±SEM of three independent experiments. *P<0.05, **P<0.01, and ****P<0.0001 versus DOX alone.

We then hypothesized whether MTP18 relies on DRP1 to exert its mitochondrial fragmentation and pro-apoptotic effects. To this end, we conducted a co-expression model using stable MTP18 overexpressed AGS cell line transfected with DRP1-siRNA. Then, we exposed different groups of cells to DOX. Our results showed that overexpression of MTP18 significantly increased DOX-induced mitochondrial fission and apoptosis. However, when DRP1 was knockdown in the presence of MTP18 overexpression, there was a significant reduction in percentages of cells undergoing mitochondrial fission (Figure 7A) and apoptosis upon high dose DOX exposure (Figure 7B), suggesting that MTP18 could not exert its pro-mitochondrial fission and pro-apoptotic effect when DRP1 is poorly expressed. These data suggest that MTP18-mediated mitochondrial fission and apoptosis is dependent on DRP1.

### Fluorouracil treatment induces mitochondrial fission and apoptosis, and shows a similar trend of changes in MTP18 expression as doxorubicin exposure

**Figure 8:**
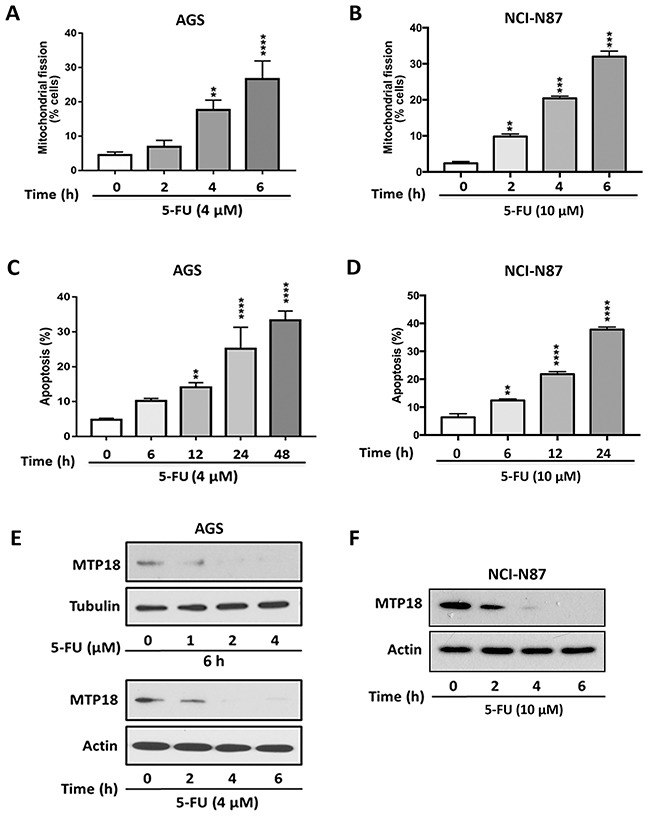
Fluorouracil treatment induces mitochondrial fission and apoptosis, and shows a similar trend of changes in MTP18 expression as doxorubicin exposure **(A)** and **(B)** Fluorouracil (5-FU) induces mitochondrial fission in gastric cancer cells. **(C)** and **(D)** 5-FU induces apoptosis in gastric cancer cells. The cells were stimulated with 5-FU (4μmol/L) in AGS cell lines (A) and (C) or (10 μmol/L) in NCI-N87 cell lines (B) and (D) and then harvested at the indicated time for mitochondrial fission and apoptosis analysis. Percentage of apoptosis was analyzed by DNA fragmentation using the cell death detection ELISA. Data were expressed as the mean±SEM of three independent experiments. **P<0.01, ***P<0.001, and ****P<0.0001 compared to non-treated control. **(E)** and **(F)** 5-FU down regulates MTP18 expression in gastric cancer cells. (E) and (F) Show kinetics of MTP18 expression. AGS (E) and NCI-N87 (F) cell lines were stimulated with indicated dose of 5-FU and then harvested at the indicated time for immunoblotting. β-actin or tubulin served as a loading control.

In order to determine the generalizability of MTP18′s role in chemotherapy induced mitochondrial fission and apoptosis in gastric cancer, we included additional experiments using Fluorouracil (5-FU), which is considered a first line chemotherapeutic agent for advanced gastrointestinal cancers [37, 38], and tested its effects in an additional gastric cancer NCI-N87 cell line. We detected whether 5-FU induced mitochondrial fission and apoptosis in both AGS and NCI-N87 cell lines. We observed 5-FU induced mitochondrial fission (Figure 8A and 8B) and apoptosis (Figure 8C and 8D) in both gastric cancer cell lines. Similar to results on Dox exposure, 5-FU also down regulated the MTP18 expression in both gastric cancer cell lines (Figure 8E and 8F, and Supplementary Figure 4A and 4B). These findings suggest that MTP18 may play a similar role in 5-FU induced apoptotic pathway in gastric cancer cells.

## DISCUSSION

Gastric cancer is a leading cause of cancer-related mortality worldwide. Survival has been enhanced through a combination of surgery and chemotherapy, both in the adjuvant and neoadjuvant setting. Unfortunately, treatment with curative intent is not possible with chemotherapy alone, and in the metastatic setting, chemotherapy is a palliative strategy due to the development of chemoresistance. Extensive research efforts have been made to better understand the molecular mechanism of chemoresistance in recent years. Increasing evidence has shown that mitochondrial morphology plays a crucial role in tumorigenesis and chemotherapy. Mitochondrial fusion and fission participate in the regulation of apoptosis. Mitochondrial fission is able to initiate apoptosis, in contrast, mitochondrial fusion can prevent it [22, 23]. Failure of cells to undergo efficient mitochondrial fission is correlated with the resistance to apoptotic cell death [39–41]. Anthracycline chemotherapeutics are critical to the treatment of gastric cancer, including doxorubicin (DOX), mediate their apoptotic signal via regulation of mitochondrial fission machinery [14, 42]. Therefore, in order to identify mediators of chemo-induced fission and targets of chemoresistance, we endeavored to determine the molecular mechanism of DOX treatment in mitochondria. This study identifies a novel target molecule MTP18 that enhances the sensitivity of gastric cancer cells to chemotherapy through regulation of mitochondrial fission. Our data showed that over expression of MTP18 enhanced the sensitivity of gastric cancer cells to DOX-induced apoptosis by promoting DRP1 mediated-mitochondrial fission. MTP18-enhanced chemosensitivity is abolished when DRP1 is knockdown. Therefore, this study proposed a novel signaling pathway in DOX-induced apoptosis that is mediated by MTP18 and DRP1 within mitochondria.

MTP18 is a novel PI-3 kinase dependent expression protein. It is predicted to localize in mitochondrial inner membrane in mammalian cells [29]. MTP18 is responsible for maintaining mitochondrial morphology and viability in various cancer cell lines [28, 29]. Our results showed that DOX induced down-regulation of MTP18 expression in gastric cancer cells, suggesting that MTP18 might have mechanistic correlation with DOX resistance in gastric cancer. Previous studies showed that inhibition of MTP18 expression led to excessive mitochondrial fusion; whereas, excessive mitochondrial fission was noted when MTP18 overexpressed in different cancer cell lines [28, 29]. Consistent to previous findings, our results in gastric cancer AGS cells also showed that knockdown of endogenous MTP18 expression significantly reduced mitochondrial fission. Although MTP18 exhibits pro-mitochondrial fission effect in different cancer cell lines, its effects on apoptosis among different cancer cell types remain inconclusive. Previous studies in PC-3, HeLa, COS-7 and HeCAT cells found that both MTP18-knockdown and overexpressed groups resulted in increased apoptosis [28, 29]. This could be related to the breakage of mitochondrial dynamic due to excessive fission in the state of MTP18 overexpression, or extensive fusion upon knocking down of MTP18. Both situations triggered the release of cytochrome c and resulted in activation of downstream apoptotic cascade [43]. In contrast, our data showed that DOX-induced apoptosis in gastric cancer cells significantly decreased when MTP18 was knocked down. Conversely, when MTP18 was overexpressed, even a low concentration of DOX could sensitize a significant number of gastric cancer cells to undergo apoptosis. These findings suggest that MTP18 is mainly pro-mitochondrial fission and pro-apoptotic in gastric cancer cells.

It has been reported that MTP18 is actively involved in setting up the fission complex inside the mitochondrion [28, 40, 44]. However, very few reports have been made on the role of MTP18 in chemotherapy-induced apoptosis. To understand how MTP18 is mechanistically regulating mitochondrial morphology, we performed the target protein interaction prediction with stringv10, on which DRP1 scored the highest among all the possible target proteins. Our data, however, showed that modulation of MTP18 expression did not significantly effect DRP1 expression. DRP1, also known as DNM1L, is a cytoplasmic GTPase. Recruitment of DRP1 to the mitochondrial outer membrane mediates the scission of the outer membrane, resulting in mitochondrial fission [33, 45]. Fragmentation of mitochondria increases the membrane permeability, which leads to the release of cytochrome-c to cytosol [45–47]. DRP1 GTPase domain is responsible for the regulation of mitochondrial morphology; cells transfected with mutated DRP1 GTPase domain suffered a profound alteration in mitochondrial morphology and disturbed the even distribution of mitochondrial tubules throughout the cells [48]. Nonetheless, DRP1 overexpression itself is not sufficient to drive the division of both membranes [26–28]. Additionally, DRP1 also serves as the downstream mediator of P53 conveying its apoptotic signal. p53 transcriptionally activates DRP1 and initiates the mitochondrial apoptotic pathway [32].

Here, we observed that DOX required DRP1 to induce mitochondrial fission and subsequent apoptosis. Intriguingly, our data showed that MTP18 can affect the DRP1 translocation to mitochondria during DOX exposure. Our subcellular fraction analysis and IP data suggest that MTP18 might associate directly with DRP1 at the mitochondrial membrane to induce fission of mitochondrial membrane during the early phase of DOX exposure. Taken together, our data clearly showed that MTP18 could be one of the essential inner membrane components for the initiation of mitochondrial fission machinery. Downregulation of MTP18 expression during DOX exposure could be one of the resistance factors interfering with chemotherapy-induced apoptosis in gastric cancer cells.

In summary, this study suggests a pro-apoptotic function of mitochondrial protein MTP18 in gastric cancer. MTP18 enhances gastric cancer cells’ chemosensitivity by promoting apoptosis through DRP1-mediated mitochondrial fission. Hence, MTP18 may have a potential therapeutic role in gastric cancer management. However, further *in vitro* and *in vivo* studies are required to elucidate the mechanism of MTP18-induced chemosenstivity and to confirm whether the pro-apoptotic effect of MTP18 is reproducible, as well as to explore its potential to be translated into clinical value.

## MATERIALS AND METHODS

### Cell cultures and doxorubicin treatment

Human gastric cancer cell lines AGS and NCI-N87 were purchased from American Type Culture Collection (ATCC, Manassas, VA, USA). The AGS cells were grown in Dulbecco’s Modified Eagle Medium Nutrient Mixture F12 (DMEM/F12; Gibco, UK) and NCI-N87 cells were in RPMI-1640 media (Gibco, UK), supplemented with 10% fetal bovine serum, and 100 U/mL penicillin, and 100 μg/mL streptomycin (Gibco, UK) in a humidified atmosphere of 5% CO2 at 37°C [49]. Doxorubicin and was purchased from Sigma-Aldrich (St. Louis, MO, USA). The treatment with doxorubicin was performed as we described [16].

### Lentiviral construct of MTP18-shRNA and infection

MTP18-shRNA and control-shRNA lentiviral particles were purchase from Santa Cruz Biotechnology, Inc. (Dallas, TX, USA). MTP18-shRNA contains a pool of concentrated, transduction ready viral particles containing 3 target-specific constructs, encoding shRNA designed to knockdown MTP18 expression and control shRNA lentiviral particles contains an shRNA construct encoding a scramble sequence that will not lead to specific degradation of any know cellular mRNA. The MTP18 shRNA: sc-75842-VA hairpin sequence was 5′-GATCCCTCCTCCTGATCATACTCTTTCAAGAGAAGAGTATGATCAGGAGGAGTTTTT; sc-75842-VB hairpin sequence was GATCCGACAGAAGCTTAGAGACAATTCAA GAGAT TGTCTCTAAGCTTCTGT TTTTT; and sc-75842-VC hairpin sequence was GATCCCAAGGAATAGGCC CAAGATTTCAAGAG AATCTTGGGCCTATTCCTTG TTTTT. The scramble MTP18 shRNA sense seq-uence was 5′-GCACTACCAGAG CTAACTCAGATAGTACT-3′, and the antisense sequence was 5′-AGTACTATCTGAG TTAGCTCTGGTAGTGC-3′. Cells were infected with the virus according to manufactural protocol. The stable transfected clones were selected in a culture medium containing 2.5 μg/mL puromycin (Life Technologies, Grand Island, NY, USA) for 4 weeks. The stable transfected cells were seeded into 96-well plate by limited dilution in order to obtain single cell colony. Several single cell colonies were cultured and the MTP18 expression levels were checked by western blot.

### Lenti ORF clone of MTP18 construct and infection

Lentiviral particle harboring the cDNA of MTP18 was purchased from Origene (Rockville, MD, USA). Viruses were amplified in 293T cells. Cells were infected with the virus according to manufactural protocol. The stable transfected clones were selected in a culture medium containing 1.0 mg/mL Geneticin (Life Technologies, Grand Island, NY, USA) for 4 weeks. The stable transfected cells were seeded into 96-well plate by limited dilution in order to obtain single cell colony. Several single cell colonies were cultured and the MTP18 expression levels were checked by western blot.

### DRP1 siRNA transfection

The DRP1 siRNA (h) and control siRNA-A (scramble siRNA) were purchased from Santa Cruz Biotechnology, Inc. (Dallas, TX, USA). DRP1 siRNA (h) is a pool of three target-specific 19-25 nt siRNAs designed to knock down DRP1 gene expression. For DRP1 siRNA (h), the sense sequence is 5′-AACGCAGAGCAGCGGAAAGAGtt-3′ and antisense is 5′CUCU UUCCGCUGCUCUG CGU Utt-3′. For scramble siRNA, the sense sequence is 5′-UUCUCCGAACGUGUCACGUtt-3′ and antisense 5′-ACGUGACACGUUCGGAGAAtt-3′ sequence is Cells were seeded 24 hours before transfection. Cells were then transfected with 50 nM scramble siRNA or DRP1 siRNA (h) using Lipofectamine® 2000 Reagent (Invitrogen, Carlsbad, CA, USA) according to the manufacturer’s instructions.

### Immunoblotting

Immunoblotting was performed as we reported earlier [29]. In brief, cells were lysed for 1h at 4°C in RIPA buffer [20 mmol/L Tris (pH 7.5), 2 mmol/L EDTA, 3 mmol/L EGTA, 2 mmol/L DTT, 250 mmol/L sucrose, 0.1 mmol/L phenylmethylsulfonyl fluoride (PMSF), 1% Triton X-100 and a protease inhibitor cocktail]. The samples were separated by sodium dodecyl sulfate–PAGE (SDS–PAGE) using a 4-20% Mini-PROTEAN TGX™ Precast Gel (Bio-Rad, Hercules, CA, USA) and transferred 1 hour at 80V to PVDF membrane (Merck Millipore Ltd, Darmstadt, Germany). Equal-protein loading was controlled by Ponceau red staining of membranes. The membrane was blocked 5% milk in Tris buffered saline with 0.1% of Tween 20 for 1 h, and then was incubated overnight with primary antibody. Blots were then probed by horseradish peroxidase-conjugated goat anti-rabbit IgG Santa Cruz Biotechnology, Inc. (Dallas, TX, USA). Antigen-antibody complexes were detected visualized by Amersham^TM^ ECL^TM^ Prime Western Blotting Detection Reagent (GE Healthcare, Buckinghamshire, UK). Anti-MTP18, anti-caspase-3 and anti-PARP polyclonal antibodies were from Abcam. Anti-tubulin polyclonal, and anti-cyclooxygenase IV and anti-cytochrome c monoclonal antibodies were from Cell signaling Technology, Inc. (Danvers, MA, USA). Anti-actin monoclonal and Anti-DRP1 polyclonal antibodies were from Santa Cruz Biotechnology, Inc. The protein band intensity was quantified by ImageJ (National Institutes of Health, Bethesda, Maryland, USA) using protocol written by Luke Miller, November 2010 (http://www.lukemiller.org/ImageJ_gel_analysis.pdf). Briefly, the density of each sample was first quantified with image J, then the percent value of each sample and that of standard was calculated. Finally, the relative density was calculated by dividing the percent value of each sample by the percent value of each standard.

### Immunoprecipitation

Immunoprecipitation was carried out as we described [50]. In brief, cells were lysed for 1 h at 4°C in a lysis buffer. To perform immunoprecipitation, the cell lysates were precleared with 10% (vol/vol) protein A-agarose (Roche, Branford, CT, USA) for 1 h on a rocking platform. Specific antibodies were added and rocked for overnight. Immunoprecipitates were captured with 10% (vol/vol) protein A-agarose for another hour. The agarose beads were spun down and washed thrice with NET buffer. The antigens were released and denatured by adding SDS sample buffer.

### DNA fragmentation and apoptosis assays

DNA fragmentation was monitored using the cell death detection ELISA kit (Roche, Branford, CT, USA) as we have described elsewhere [50]. Briefly, the anti-histone monoclonal antibody was added to the 96 well ELISA plates and incubated overnight at 4°C. After recoating and three rinses, the cytoplasmic fractions were added and incubated for 90 min at room temperature. After three washes, bound nucleosomes were detected by the addition of anti-DNA peroxidase monoclonal antibody and reacted for 90 min at room temperature. After the addition of the substrate, the optical density was determined at 405 nm using an ELISA reader. For apoptosis analysis, a terminal deoxynucleotidyl transferase-mediated Dutp nick-end-labeling (TUNEL) kit (Clontech, Mountain View, CA, USA) was used. After transfection and treatment as indicated, cells were rinsed, fixed, permeabilized and stained with the TUNEL assay according to the kit’s instructions. Images were taken using a laser scanning confocal microscope (Zeiss LSM 710 BIG, Dublin, CA, USA). One hundred fifty to two hundred cells were counted in 20-30 random fields in each group. Results are expressed as percentage of TUNEL positive cells.

### Preparation of mitochondrial fractions

Mitochondrial fractions were prepared as we described [50]. Briefly, cells were washed twice with PBS and the pellet was suspended in 0.2 ml of buffer A (20 mM HEPES pH 7.5, 10 mM KCl, 1.5 mM MgCl_2_, 1 mM EGTA, 1 mM EDTA, 1 mM DTT, 0.1 mM PMSF, 250 mM sucrose) containing a protease inhibitor cocktail (Sigma-Aldrich, St. Louis, MO, USA). The cells were homogenized by 12 strokes in a Dounce homogenizer. The homogenates were centrifuged twice at 750 g for 5 min at 4°C to collect nuclei and debris. The supernatants were centrifuged at 10000 g for 15 min at 4°C to collect mitochondria-enriched heavy membranes (HM). The resulting supernatants were centrifuged to yield cytosolic fractions.

### Analysis of mitochondrial fission

Mitochondrial fission was analyzed by staining mitochondria as we and others have described earlier with some modification [16, 45]. Briefly, cells were plated onto the coverslips. After treatment, they were stained for 15 min with 100 nM MitoTracker Red CMXRos (Molecular Probes, Eugene, OR, USA). Cells were fixed in 4% paraformaldehyde for 15 minutes, permeabilized with 0.2% Triton X-100. Mitochondria were imaged using a laser scanning confocal microscope (Zeiss LSM 710 BIG, Dublin, USA). To quantitatively analyze cells with mitochondria fission, those cells with disintegrated mitochondria were taken as mitochondrial fission. The percentage of cells with fragmented mitochondria relative to the total number of cells was presented as the mean ± SEM of at least three independent experiments, counted by an observer blinded to the experimental conditions. 100-150 cells in 20-30 random fields were counted.

### Prediction of a potential MTP18′s target protein

The potential target protein was predicted using string v10 (http://string-db.org/cgi/input.pl). The search term was set as “MTP18 or MTFP1” and organism as “Homo sapiens”. The protein-protein interaction was determined by the interaction score. It is an indicator of confidence regarding how likely STRING judges an interaction to be true, given the available evidence. The score can be ranged from 0 to 1, with 1 being the highest possible confidence [51].

### Statistical analysis

Data are expressed as the mean ± SEM of at least three independent experiments for each experimental group. We evaluated the data with Student’s t test. We used a one-way analysis of variance for multiple comparisons. A value of *P*<0.05 was considered significant.

## SUPPLEMENTARY MATERIALS FIGURES AND TABLES



## Abbreviations

MTP18 or MTFP1: Mitochondrial Protein 18 or Mitochondrial Fission Protein 1
DRP1 or DNM1L: Dynamin Related Protein 1 or Dynamin 1-Like Protein
DOX: Doxorubicin
ELISA: Enzyme-Linked ImmunoSorbent Assay
TUNEL: Terminal deoxyucleotidyl transferase dUTP Nick End Labeling
siRNA: Small (or short) Interfering RNA
shRNA: Short Hairpin RNA or Small Hairpin RNA
cDNA: Complementary DNA
5-FU: Fluorouracil
AGS: Gastric Adenocarcinoma
SEM: Standard Error of the Mean
STRING: Search Tool for Recurring Instances of Neighbouring Genes
