# Supplementary material for: Mitochondrial protein 18 (MTP18) plays a pro-apoptotic role in chemotherapy-induced gastric cancer cell apoptosis
Source: Oncotarget. 2017 Apr 28;8(34):56582–97. doi: 10.18632/oncotarget.17508 (PMC5593585; doi:10.18632/oncotarget.17508)
Supplement: Supplementary file 1 [file oncotarget-08-56582-s001.pdf]

Mitochondrial protein 18 (MTP18) plays a pro-apoptotic role in chemotherapy-induced gastric cancer cell apoptosis

SUPPLEMENTARY MATERIALS

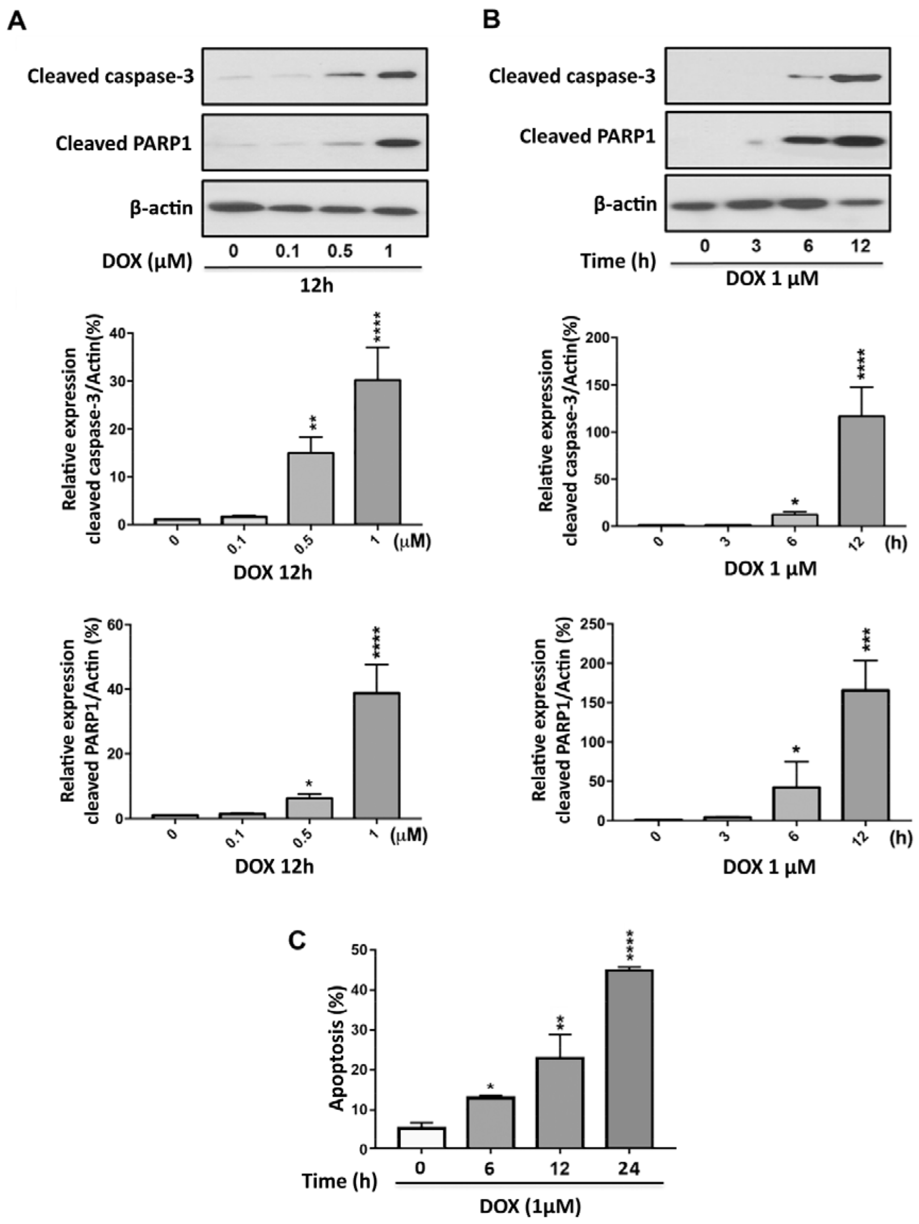

Supplementary Figure 1: Doxorubicin induces apoptosis in gastric cancer cells. **(A)** and **(B)** Doxorubicin (DOX) exposure induces caspase-3 and PARP1 cleavage. **(A)** AGS cells were stimulated with the indicated doses of DOX, and harvested after 12h for immunoblotting. **(B)** AGS cells were stimulated with 1μmol/L DOX and then harvested at the indicated time for immunoblotting. **(A)** and **(B)** *(up panels)* Show caspase-3 and PARP1 cleavage. β-actin served as a loading control. **(A)** and **(B)** (*low panels*) Show densitometry analysis. The densitometry data were expressed as the mean±SEM of three independent experiments. **(C)** AGS cells were stimulated with 1μmol/L DOX and then harvested at the indicated time for detection of apoptosis related DNA fragmentation using the cell death detection ELISA. **(C)** shows percentage of apoptosis related DNA fragmentation. Data were expressed as the mean±SEM of three independent experiments. *$P<0.05$, **$P<0.01$, ***$P<0.001$, and ****$P<0.0001$ compared to non-treated control.

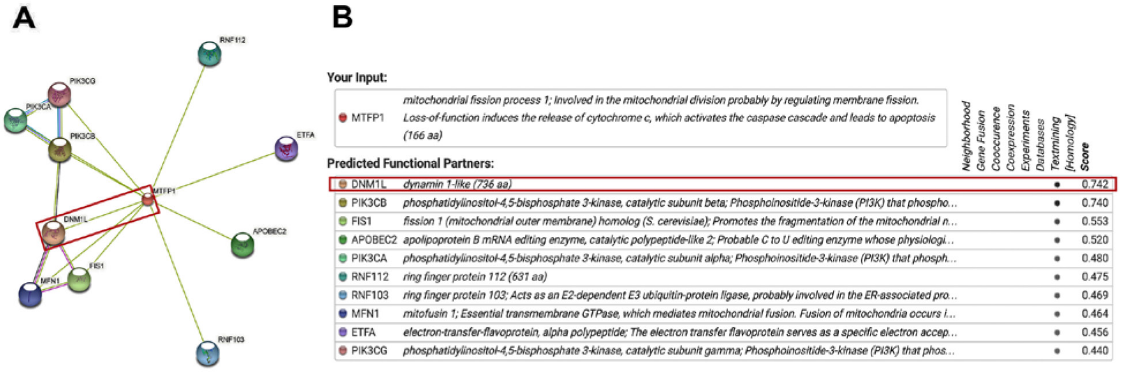

Supplementary Figure 2: DRP1 (DNM1L) was predicted to be the MTP18 (MTFP1)'s target protein. Analysis of protein interaction using String v10 (http://string.embl.de/). **(A)** shows schematic representation of target protein interaction network of MTP18 (also known as MTFP1). **(B)** Shows the confidence interaction scores of potential functionally associated proteins. DRP1 (alternative name DNM1L) shows highest estimated confidence score among all of the potential functionally associated proteins. DRP1 or DNM1L= dynamic-related protein 1 or dynamin 1-like protein; MTP18 or MTFP1= mitochondrial protein 18 or mitochondrial fission protein 1.

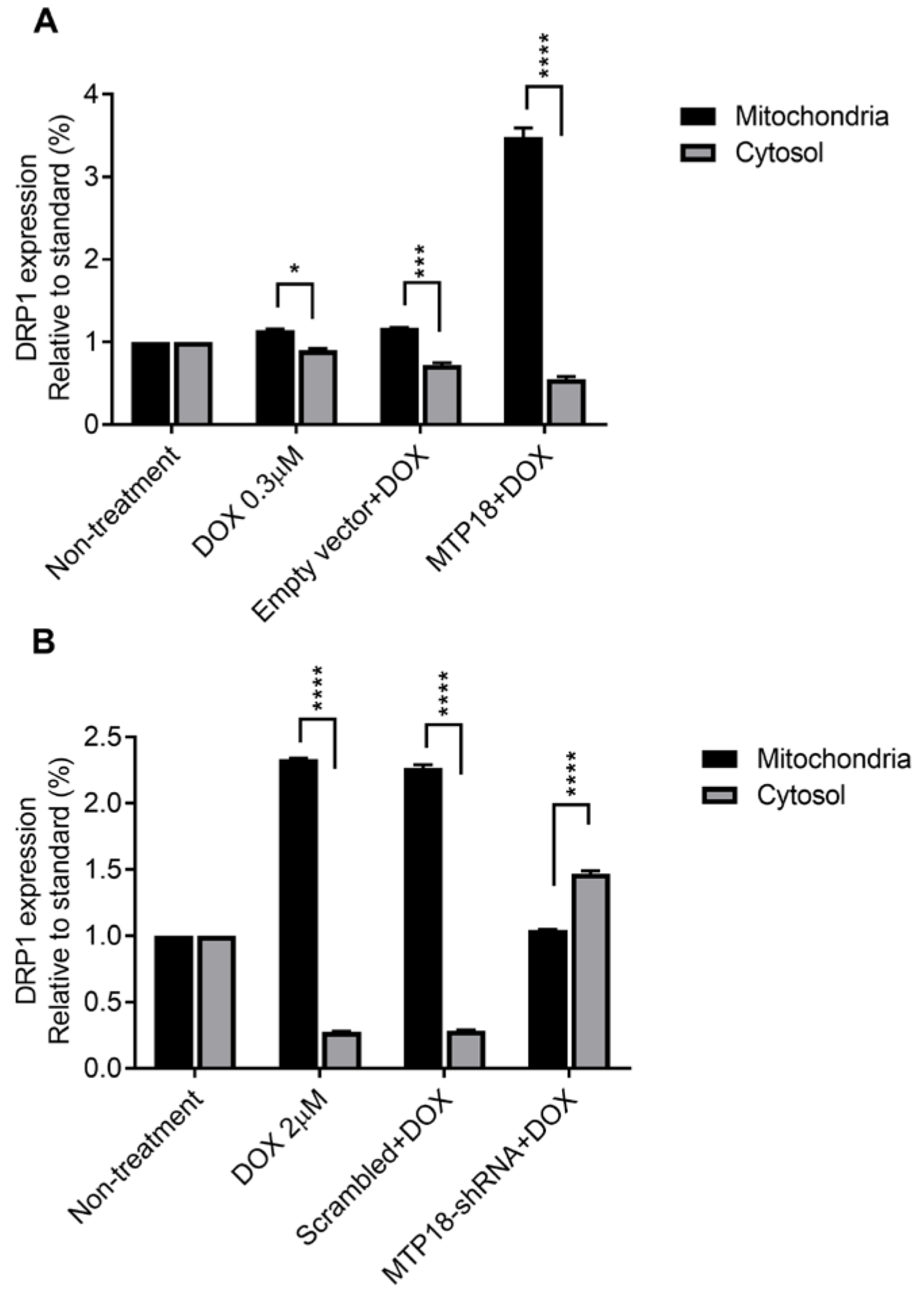

Supplementary Figure 3: MTP18 promotes doxorubicin-induced DRP1 accumulation in mitochondria. **(A)** and **(B)** Densitometry analysis of DRP1 expression in mitochondria and cytosolic fraction. **(A)** Shows an increase in DRP1 accumulation in mitochondria upon enforced expression of MTP18 (correspond to immunoblot data of Figure 6C). **(B)** Shows a reduction in DRP1 accumulation when MTP18 was knocked down (correspond to immunoblot data of Figure 6D). Cytochrome c oxidase (COXIV) served as a loading control for mitochondria-enriched heavy membranes (HM) and β-actin served as a loading control for cytosolic fraction. Data were expressed as the mean±SEM of three independent experiments. **P*<0.05, ****P*<0.001, and *****P*<0.0001

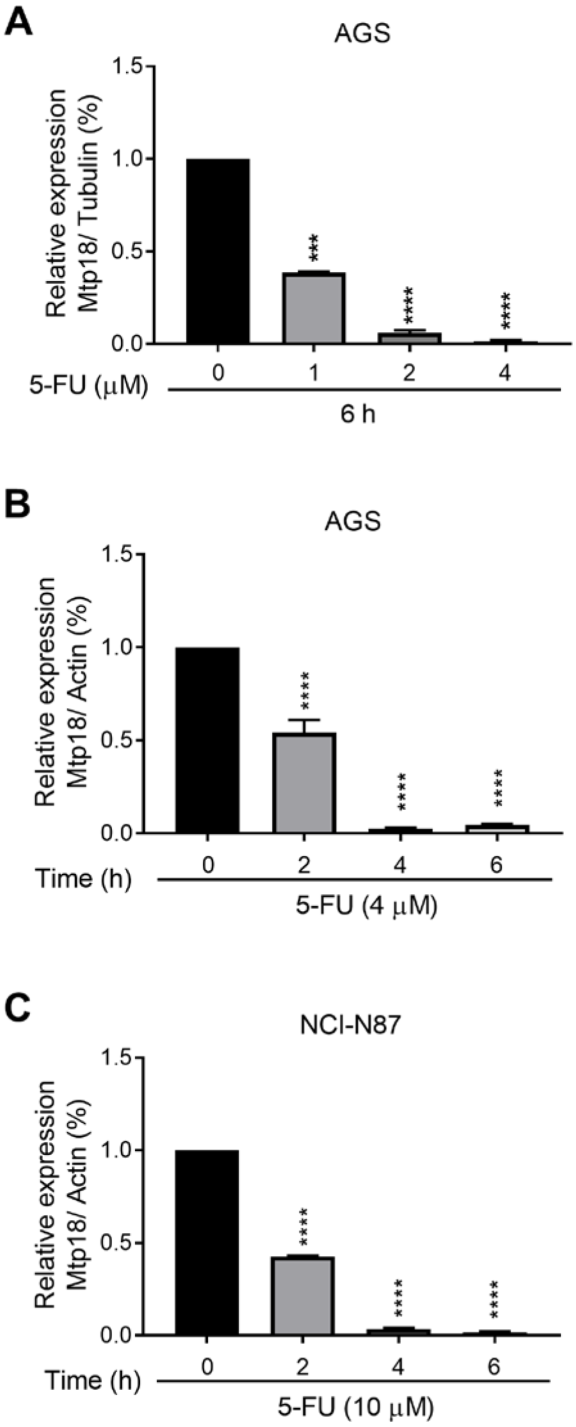

Supplementary Figure 4: Fluorouracil treatment downregulates MTP18 expression. (A-C) Densitometry analysis of MTP18 expression. **(A)** and **(B)** Show kinetics of MTP18 expression in AGS cell line (correspond to immunoblot data of Figure 8E) and **(C)** shows kinetics of MTP18 expression in NCI-N87 cell line (correspond to immunoblot data of Figure 8F) in response to 5-FU exposure. Cells were stimulated with indicated dose of fluorouracil (5-FU) and then harvested at the indicated time for immunoblotting. β-actin or tubulin served as a loading control. Data were expressed as the mean±SEM of three independent experiments. ****P*<0.001, and *****P*<0.0001 compared to non-treated.
